# Development of a Machine Learning Algorithm for the Prediction of WHO Grade 1 Meningioma Recurrence

**DOI:** 10.7759/cureus.82033

**Published:** 2025-04-10

**Authors:** Simon G Ammanuel, Matthew Stenerson, Thomas Staniszewski, Manasa Kalluri, Benjamin Lee, Elsa Nico, Azam S Ahmed

**Affiliations:** 1 Department of Neurological Surgery, University of Wisconsin Hospitals and Clinics, Madison, USA; 2 Department of Neurological Surgery, University of Wisconsin School of Medicine and Public Health, Madison, USA

**Keywords:** artificial intelligence, grade 1 meningioma, machine learning, meningioma, neurosurgery

## Abstract

Objective

Meningiomas commonly recur following gross total resection (GTR), and the risk of recurrence is difficult to predict using current classification schemes such as the World Health Organization (WHO) tumor grade. This study aimed to create a predictive model of recurrence risk following GTR of WHO grade 1 meningiomas based on histopathological and epidemiological factors.

Methods

A retrospective chart review was completed for all patients at our institution who underwent their first surgery for a WHO grade 1 meningioma between 2017 and 2022. Those with genetic predispositions, such as neurofibromatosis type 2, were excluded. Baseline characteristics, including histopathology findings, were obtained, and we used a Risk-calibrated Superspase Linear Integer Model (Risk-SLIM) with a five-fold cross-validation (CV) to create a predictive model of recurrence over an average follow-up of three years.

Results

Univariate analysis of our selected variables revealed a significant predictive association between WHO grade 1 meningioma recurrence and subtotal resection but not with any other variable. However, the meningioma recurrence score (MRS) generated by our machine learning algorithm revealed multiple predictive factors of recurrence, including age, female gender, and various histopathologic features, including the Ki-67/MIB-1 index.

Conclusions

Machine learning algorithms like the one we present here may help identify patients at high risk of recurrence of their WHO grade 1 meningioma, and they are more likely to benefit from closer postoperative surveillance or adjuvant treatment, even when GTR is achieved.

## Introduction

Meningiomas represent the most common primary tumor of the adult central nervous system (CNS), with an estimated annual incidence in the United States of approximately 8.6 per 100,000 people and a median age at diagnosis of 66 years [1,2]. Among all meningiomas discovered on neuroimaging, approximately 32-39% are asymptomatic, and approximately 3% of all neuro-autopsies performed on patients over the age of 60 years identify a meningioma; therefore, the true incidence is likely underestimated [3,4]. The most common presenting symptoms of meningiomas include cerebral dysfunction (48.5%), headache (48.2%), cranial nerve deficits (38.9%), motor changes (30.7%), and seizures (23.6%). Importantly, initial symptoms are often nonspecific, which may lead to delayed neuroimaging [5]. The World Health Organization (WHO) 2021 Classification of CNS tumors classifies meningiomas into grades 1, 2, and 3, which define approximately 70%, 28%, and 3% of meningiomas, respectively, and are associated with estimated 10-year relative survival rates of 96.8%, 90.2%, and 30.4%, respectively [6].

Surgical resection is generally indicated for symptomatic meningiomas or those whose location could make resection treacherous if they were to grow. Radiation therapy is commonly pursued adjuvantly when histological margins are positive or as the primary treatment when surgical resection is contraindicated. Historically, the completeness of the initial surgical excision has been recognized as the strongest predictor of meningioma recurrence. In 1957, Simpson reported 10-year recurrence rates after grade 1, 2, and 3 resections of 9%, 19%, and 29%, respectively, bringing focus to the degree of resection [7]. Following advances in imaging, adjuvant therapies, such as stereotactic radiosurgery, and molecular profiling of meningiomas, the Simpson grade has mainly been supplanted by other outcome measures; however, the extent of tumor resection and the volume of residual tumor remain significant predictors of long-term outcomes [8].

More recent reports that have considered WHO grade have shown that, among patients undergoing radiation therapy or surgical resection, recurrence rates for WHO grade 1 and 2 meningiomas are approximately 0.00-2.36 and 7.35-11.46 per 100-person-years, respectively [9]. Even among patients with WHO grade 1 meningiomas status post histologically demonstrated gross total resection, approximately 20-39% will recur within 10 years [10]. Thus, there remains a need to consider individual predictors of recurrence beyond WHO grade to help distinguish patients who are likely to benefit from closer surveillance or adjuvant treatment from those at risk of overtreatment.

It has long been observed that meningiomas occur at a female-to-male ratio of approximately 3:2 and may undergo rapid growth during pregnancy, suggesting possible hormonal influences in its pathogenesis [11]. One meta-analysis from 2013 showed associations between meningioma and hormone replacement therapy, post-menopause, and parity [12]. However, large-scale epidemiological studies have produced equivocal findings on the relationship between the biological behavior of meningiomas and their expression of such receptors as sex steroid hormones and vascular endothelial growth factor receptors, among others [13].

Despite our growing understanding of tumor biology through gene expression and biomarkers, classifying meningiomas has historically relied on histological appearance. As a result, retrospective studies on meningioma outcomes are primarily limited to the histopathologic features that have been the standard clinical practice to document such as the presence of peritumoral edema, nuclear atypia, such as macronuclei, sheeting, small cells, psammoma bodies, bone invasion, and the measured Ki-67/MIB-1 labeling index [14-16]. The multifactorial nature of meningioma recurrence risk and inherent subjectivity in histological characterization make it challenging to apply these data to individual patients or in decision-making regarding their treatment and post-treatment surveillance. Thus, a machine learning algorithm that integrates these evidence-based yet disparate predictive factors may be of clinical value.

We sought to develop an algorithm to predict the risk of recurrence of WHO grade 1 meningioma based on age, sex, history of radiation therapy, and the previously mentioned histopathologic features, which neuropathologists at our institution have a standard for documenting. Our objective in developing such an algorithm was to shed light on some of the proper granularity among meningiomas classified as WHO grade 1 and better understand which factors may increase one’s risk of recurrence. 

## Materials and methods

Following institutional board review approval, a retrospective chart review was conducted of all patients presenting with cranial meningioma who underwent surgery between 2017 and 2022. Individual charts were then reviewed to identify relevant patients and extract operative and histopathologic characteristics. Histopathologic characteristics were collected from patients’ postoperative pathology reports. Patients with WHO grade 2 and grade 3 meningiomas and those with a history of genetic variants that increase the probability of developing meningiomas, such as neurofibromatosis type 2, were removed from this study. Variables were selected given their association with recurrence in prior studies and the frequency at which neuropathologists reported them at our institution. Recurrence was determined at routine outpatient follow-up visits with advanced imaging, with magnetic resonance imaging (MRI) showing regrowth of the tumor near the surgical lesion. Patients missing any of the previously mentioned variables were excluded. Variables were stratified categorically to simplify the prediction model and increase the effect size for each factor. Continuous and categorical variables were initially analyzed using either the Mann-Whitney U test or Fisher’s exact test. Statistical significance was deemed at p-values of <0.05 for each test.

A new modeling method known as the Risk-Calibrated Superspase Linear Integer Model (Risk-SLIM) was created to reduce the variability in risk assessment using machine learning techniques. This technique created an optimized risk score with superior risk calibration using an optimized area under the curve (AUC). The AUC is a score to identify the performance of the model. The Risk-SLIM method has been used in various risk prediction models and has been shown to produce excellent results with external validation. Using this method, a risk stratification score was created to identify patients at high risk for WHO grade 1 meningioma recurrence. To assess risk validation, we also examined the average calibration error (CAL), the mean-squared error between the predicted probability and the observed score. The CAL exhibits how close the model score is to the observed score. Following the creation of our model, a five-fold cross-validation (CV) was utilized. Specifically, the data were randomly split into five separate parts, where the model was trained using four of the five folds, and then the model was tested using the last fold of the model. These were denoted as five-fold CV AUC and five-fold CV CAL errors, respectively. A receiver operating characteristic (ROC) curve was conducted to evaluate the predictive ability of the proposed model. All statistics and models were conducted in Python version 3.10 (Python Software Foundation, Wilmington, Delaware, US).

## Results

Following individual chart review, 250 patients were identified as undergoing resection for meningiomas between 2017 and 2022, with an average follow-up time of 3.01 years. Of these, 13 (5.2%) patients experienced recurrence at an average follow-up of 6.88 years, and 3 (1.2%) patients experienced 2 or more recurrences 14-24 months after their second resection. A univariate analysis of the clinical, operative, and histopathologic characteristics between the non-recurrence and recurrence groups was conducted following the chart review. For univariate analysis, gross total resection was significantly associated with non-recurrence, with p = 0.006. Ki-67/MIB-1 index > 5% was trending toward a significant association with recurrence (p = 0.07). No significant difference was noted for any other patient, tumor, or histopathologic feature (p > 0.05) (Table 1).

**Table 1:** Patient, radiographic, and operative characteristics between two groups Significant values are in bold (P<0.05).

Variable	No Recurrence (n=237)	Recurrence (n=13)	P-value
Age (years), mean ±SE	60.5 ± 0.79	61.8 ± 3.79	0.72
Female sex	175 (73.8%)	8 (61.5%)	0.34
Infratentorial location	22 (9.3%)	1 (7.7%)	1.00
Peritumoral edema	72 (30.4%)	6 (46.2%)	0.23
Gross total resection	206 (86.9%)	7 (53.8%)	0.006
Sheeting	20 (8.4%)	1 (7.7%)	1.00
Necrosis	21 (8.9%)	2 (15.4 %)	0.34
Hypercellularity	60 (25.3%)	3 (23.1 %)	1.00
Small cells	10 (4.2%)	1 (7.7%)	0.45
Macronucleoli	23 (9.7%)	2 (15.4%)	0.63
Bone invasion	12 (5.1%)	2 (15.4%)	0.16
Psammomatous calcifications	56 (23.6%)	2 (15.4%)	0.74
Ki-67/MIB > 5%	7 (3.0%)	2 (15.4%)	0.07

**Figure 1:**
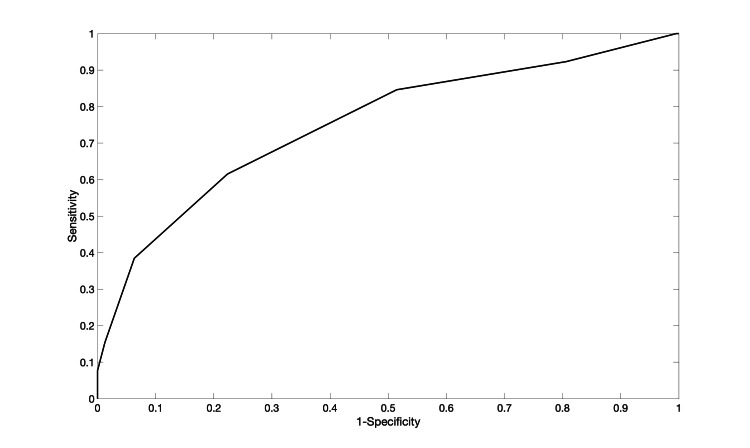
ROC curve for the Risk-SLIM model with a fivefold cross-validation AUC of 0.76 and fivefold cross-validation calibration error of 4.7% ROC: receiver operating characteristic; Risk-SLIM: Risk-calibrated Superspase Linear Integer Model; AUC: area under the curve

The meningioma recurrence score (MRS) was then created using a machine learning algorithm. It had a five-fold cross-validation area under the curve of 0.76 (Figure 1), and the CAL error was 4.7%.

Predictive factors were found to include age > over 65, female gender, presence of peritumoral edema, gross total resection, Ki-67/MIB-1>5%, presence of macronuclei, bone invasion, psammomatous calcifications, sheeting, and small cells (Table 2 and Table 3).

**Table 2:** Meningioma recurrence score for evaluating recurrence risk

Variables	Point Assigned
Bone invasion	2
Age>65	1
Presence of peritumoral edema	1
Ki-67/MIB-1>5%	1
Macronuclei	1
Psammomatous calcifications	1
Female gender	-1
Sheeting	-1
Small cells	-1
Gross total resection	-2

**Table 3:** Meningioma recurrence total score corresponding to the percentage of recurrence

Total score	Percentage (%)
-5	0.09
-4	0.24
-3	0.67
-2	1.7
-1	4.7
0	11.9
1	26.9
2	50
3	73.1
4	88.1
5	95.2
6	98.2
7	99.3

## Discussion

Meningioma recurrence despite GTR remains an incompletely understood challenge for neurosurgeons, even among patients with WHO grade 1 tumors. With an increasing probability of transformation into higher-grade tumors and an overall rise in mortality and morbidity with each recurrence, identifying patients at high risk of recurrence is key to having a more informed conversation with patients and their loved ones around prognosis, surveillance, or even preemptive adjuvant treatments [17]. At the same time, available adjuvant treatments, such as radiosurgery, carry significant risks, warranting caution around potential overtreatment. Radiosurgery complications include radiation necrosis, hemorrhage, fatigue, alopecia, leukoencephalopathy, pseudoprogression, and radiation-induced tumors [18]. An individual’s risk of recurrence after GTR of a WHO grade 1 meningioma is influenced by numerous factors - clinical, histological, and molecular - challenging to integrate consistently. Machine learning algorithms are a promising means of developing more robust predictive tools for clinical use.

Our machine learning algorithm was developed to factor in histopathological features of resected WHO grade 1 meningioma that have been standard practice to document at our institution. Historically, neuropathologists have emphasized these characteristics to make the most precise diagnosis possible, but less has been understood about how these variables may hold predictive value about recurrence. Of the histological features we included in our analysis of WHO grade 1 meningioma, we found that recurrence was significantly associated with peritumoral edema, Ki-67/MIB-1 > 5%, macronuclei, psammoma bodies, sheeting, small cells, and bone invasion. In support of our findings, peritumoral edema is an established risk factor for meningioma recurrence [19-22]. Additionally, elevated Ki-67 and MIB-1 levels and Ki-67/MIB-1 labeling index have been associated with higher rates of meningioma recurrence in multiple other studies [23-27]. In a 2021 study of 299 resected WHO grade 1 meningiomas, Nowak-Choi et al. reported that a high Ki-67 (≥ 5 vs. < 5) was associated with more than double the increased risk of recurrence. Similarly, in 322 patients with WHO grade 1 meningioma, Prat-Acín et al. demonstrated that a Ki-67/MIB-1 index of ≥ 3 increases the recurrence rate [25]. Considering the presence or absence of these variables individually offers little clinical value. However, tools like ours that integrate these many variables may become a practical means of predicting meningioma recurrence risk.

Our algorithm has some important limitations, starting with the fact that it was developed exclusively from patients with WHO grade 1 meningiomas. Furthermore, it has not been validated among patient populations at other institutions or across more significant geographic regions. The patients in our study were followed for a limited amount of time (an average of 3.01 years); the predictive value of our algorithm would benefit from a follow-up time of at least 10 years, which would be a more rigorous benchmark for monitoring recurrence. We were unable to include the meningioma subtype, as described in Table 1, in our analysis because it was inconsistently specified in the pathology reports; future analyses that do include this subtyping could help direct focus on the subpopulations of meningiomas more likely to recur. Finally, we recognize that our algorithm does not account for all variables impacting the recurrence rate. Notably, integrating molecular markers into a predictive tool would be a priority in developing future tools once more molecular data become available. For example, in 2021, Damen et al. examined 44 patients who had undergone adjuvant radiosurgery following subtotal resection of a WHO grade 1 meningioma and found that loss of 1p36 was associated with significantly higher rates of regrowth [17]. Analysis of 1p36 deletion in meningioma is not currently standard practice, but future studies that include this variable, among other genetic markers, may help refine predictive models of recurrence.

## Conclusions

The meningioma recurrence score (MRS) efficiently predicts recurrence risk in patients with WHO grade 1 meningioma, even when surgical GTR is achieved. Validating this tool across other institutions and geographic areas and expanding it to include gene expression variables can improve discussions between providers and patients about an individual’s risk of postoperative recurrence and the risks and benefits of postoperative surveillance and adjuvant treatment options.
